# β-caryophyllene and docosahexaenoic acid, isolated or associated, have potential antinociceptive and anti-inflammatory effects in vitro and in vivo

**DOI:** 10.1038/s41598-022-23842-1

**Published:** 2022-11-10

**Authors:** Laís Ferraz Brito Sousa, Hellen Braga Martins Oliveira, Nathan das Neves Selis, Lorena Lobo Brito Morbeck, Talita Costa Santos, Lucas Santana Coelho da Silva, Jully Chayra Santos Viana, Mariane Mares Reis, Beatriz Almeida Sampaio, Guilherme Barreto Campos, Jorge Timenetsky, Regiane Yatsuda, Lucas Miranda Marques

**Affiliations:** 1 Multidisciplinary Institute of Health, Federal University of Bahia (UFBA), Rua Rio de Contas, 58 - Quadra 17 - Lote 58, Bairro Candeias, CEP: 45.029-094, Vitória da Conquista, BA Brazil; 2 University of Santa Cruz (UESC), Campus Soane Nazaré de Andrade, Ilhéus, Brazil; 3 Department of Microbiology, Institute of Biomedical Science, University of São Paulo, São Paulo, Brazil

**Keywords:** Cytokines, Infectious diseases, Cellular microbiology

## Abstract

Inflammation is a complex biological response involving the immune, autonomic, vascular, and somatosensory systems that occurs through the synthesis of inflammatory mediators and pain induction by the activation of nociceptors. *Staphylococcus aureus*, the main cause of bacteremia, is one of the most common and potent causes of inflammation in public health, with worse clinical outcomes in hospitals. Antioxidant substances have been evaluated as alternative therapeutic analgesics, antioxidants, anti-inflammatory agents, antitumor agents, and bactericides. Among these, we highlight the essential oils of aromatic plants, such as β-caryophyllene (BCP), and polyunsaturated fatty acids, such as docosahexaenoic acid (DHA). The objective of this study was to evaluate the biological activities of BCP–DHA association in in vitro and in vivo experimental models of antinociception and inflammation. To determine the anti-inflammatory effects, monocytes isolated from the peripheral blood of adult male volunteers were infected with methicillin-resistant *S. aureus* and incubated with treatment for cytokine dosage and gene expression analysis. Antinociceptive effects were observed in the three models when comparing the control (saline) and the BCP-DHA treatment groups. For this purpose, the antinociceptive effects were evaluated in animal models using the following tests: acetic acid-induced abdominal writhing, paw edema induced by formalin intraplantar injection, and von Frey hypernociception. There was a significant reduction in the GM-CSF, TNFα, IL-1, IL-6, and IL-12 levels and an increase in IL-10 levels in the BCP-DHA treatment groups, in addition to negative regulation of the expression of the genes involved in the intracellular inflammatory signaling cascade (IL-2, IL-6, IRF7, NLRP3, and TYK2) in all groups receiving treatment, regardless of the presence of infection. Statistically significant results (*p* < 0.05) were obtained in the acetic acid-induced abdominal writhing test, evaluation of paw edema, evaluation of paw flinching and licking in the formalin intraplantar injection model, and the von Frey hypernociception test. Therefore, BCP and DHA, either administered individually or combined, demonstrate potent anti-inflammatory and antinociceptive effects.

## Introduction

Inflammation is a physiological reaction to tissue injury and damage resulting from the presence of trauma, infection, foreign bodies, or immune reaction^1^, which occurs with the synthesis of inflammatory mediators and pain induction by activation and sensitization of nociceptors. Pain plays a crucial role in the body, protecting against harmful stimuli and promoting the repair of local injuries^2^. The coding and neural processing of harmful stimuli are referred to as nociception, a process that begins with the transformation of environmental stimuli (physical or chemical) into action potentials at pain receptors (nociceptors) located in peripheral nerve fibers and culminates in their transfer to the central nervous system through signal transduction^2,3^.

The largest defense mechanism is in the innate immune system, where there is invasion of microorganisms and tumor cells through the action of macrophages, neutrophils, and dendritic cells. The adaptive immune system includes the activity of more specialized cells, such as B and T lymphocytes, which act in the eradication of pathogens through the production of specific receptors and antibodies^4^. Inflaming is therefore a vital step for the body, as it contributes to the return of homeostasis. At the tissue level, inflammatory signs of pain, flushing, heat, edema, and loss of function result from cellular responses to damage^5^. In this environment, several inflammatory mediators are synthesized and secreted, and are divided into two classes: pro- and anti-inflammatory. Examples include cytokines (interferons, interleukins, and tumor necrosis factor-alpha), chemokines (monocyte chemoattractant protein-1), eicosanoids (prostaglandins and leukotrienes), and the nuclear transcription factor NF-ĸB. Interleukin 10, however, has both pro- and anti-inflammatory properties and is therefore considered to be regulatory.

One of the most common and impactful causes of inflammation in public health is *S. aureus*, a gram-positive bacterium found in the nasal microbiota of 20–40% of the human population. The rupture of mucous and cutaneous barriers through chronic diseases, skin lesions, or surgical interventions allows *S. aureus* to enter tissues and the bloodstream, causing infection^6^. This bacterium may be susceptible to the antibiotic methicillin (MSSA) or methicillin-resistant (MRSA), the latter of which was first described in 1961 and has since become the leading cause of bacteremia with highly poor clinical outcomes such as endocarditis, osteomyelitis, sepsis, septic shock, and high hospital costs^6,7^.

Since the discovery of penicillin in the 1990s, the interest in natural products has increased with regard to the inhibition of free radicals, attenuation of inflammation, and in the antibiotics sector. Simultaneously, bacterial resistance has increased due to the frequent prescription of an against non-bacterial infections and its indiscriminate and irregular use, leading to sublethal doses that allow an increase in the resistance spectrum of microorganisms^4,8^. Therefore, the choice of antibiotics to treat MRSA infections is restricted^7,9^.

Among the natural products, essential oils (OE) and volatile and strong-smelling substances present in aromatic plants have been studied in various contexts^10,11^. β-caryophyllene (BCP), a bicyclic sesquiterpene found in the OE of *Cannabis sativa*, cinnamon, clove, oregano, copaiba oil-resin, and black pepper, exhibits strong anti-endemic, anti-inflammatory, antitumor, and bactericidal properties^12^. Polyunsaturated fatty acids such as docosahexaenoic acid (DHA), an omega-3 derivative, have also been studied in the modulation of inflammation through the synthesis of bioactive mediators that act in inflammation resolution^13^. It has also been shown that BCP may have improved effects when administered with DHA; however, only the antinociceptive activity of this combination has been tested thus far in experimental models in vivo and in vitro^14^.

In a previous study, we analyzed the anti-inflammatory activity of β-caryophyllene and docosahexaenoic acid in a model of *S. aureus*-induced sepsis in Balb/C^15^ male mice. A positive effect was observed in the reduction of neutrophil migration in the carrageenan-induced peritonitis model, reduction of total and differential leukocyte counts after bacterial infection, lower neutrophil migration in the histological analysis of the kidneys and lungs, and lower bacterial load in the treated group. From this we triage the doses to be studied later. Therefore, the aim of this study was to determine the anti-inflammatory and antinociceptive activities of β-caryophyllene (BCP) and Docosahexaenoic acid (DHA), isolated and associated, in vitro and in vivo experimental models.

## Results

**Figure 1:**
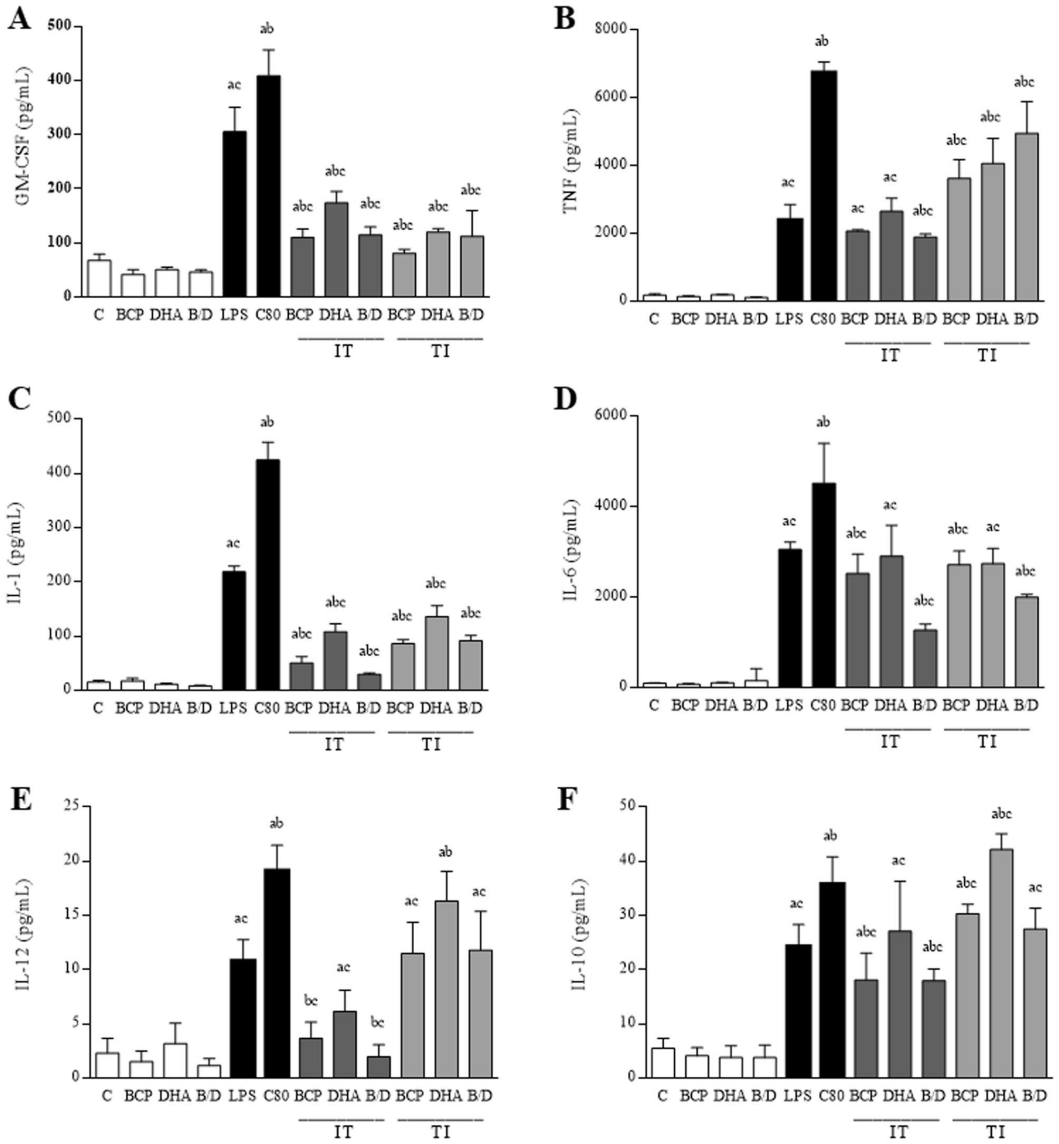
Concentration of GM-CSF (**A**), TNFα (**B**), IL-1β (**C**), IL-6 (**D**), IL-10 (**E**), and IL-12 (**F**) cytokines in the peripheral blood of male volunteers. C = control (sterile saline); BCP = beta-caryophyllene; DHA = docosahexaenoic acid; B/D = BCP and DHA association; LPS = lipopolysaccharides; C80 = strain C80; IT B = infected and treated with BCP; IT D = infected and treated with DHA; IT B/D = infected and treated with BCP and DHA association; TI B = treated with BCP and infected; TI D = treated with DHA and infected; TI B/D = treated with BCP and DHA association and infected. Data are expressed as mean ± DPM. a *p* < 0.05 vs. C; b *p* < 0.05 vs. LPS; c *p* < 0.05 vs. C80.

The volunteers were healthy and fit for the clinical trial according to laboratory analyses. The peripheral blood mononuclear cells (PBMCs) inoculated with the *S. aureus* C80 strain showed a significant increase in GM-CSF production when compared to the control group (inoculated with sterile saline, BCP, DHA, and B/D) (Fig. 1A). Both the infected and treated groups and the treated and infected groups exhibited a reduction in the expression of this cytokine when compared to the infected-only groups. Those who were first infected and later treated with BCP and DHA had a significant reduction in TNF-α expression compared to the group infected with C80, whereas the group infected and treated with BCP-DHA showed significance with regard to LPS (Fig. 1B). The group that was treated and later infected showed a TNF-α reduction in comparison to the C80 group. These treatments caused a significant reduction in IL-1β expression, and the group previously infected and treated showed a greater decrease in this cytokine (Fig. 1C). Figure 1D shows that there was a significant reduction in IL-6 expression in the treated groups compared to those infected only with C80. The groups infected and treated with BCP and with the BCP-DHA combination showed a significant decrease in IL-12 expression (Fig. 1E), whereas the highest IL-10 expression was observed in the group treated with DHA and infected with *S. aureus* (Fig. 1F).

**Figure 2:**
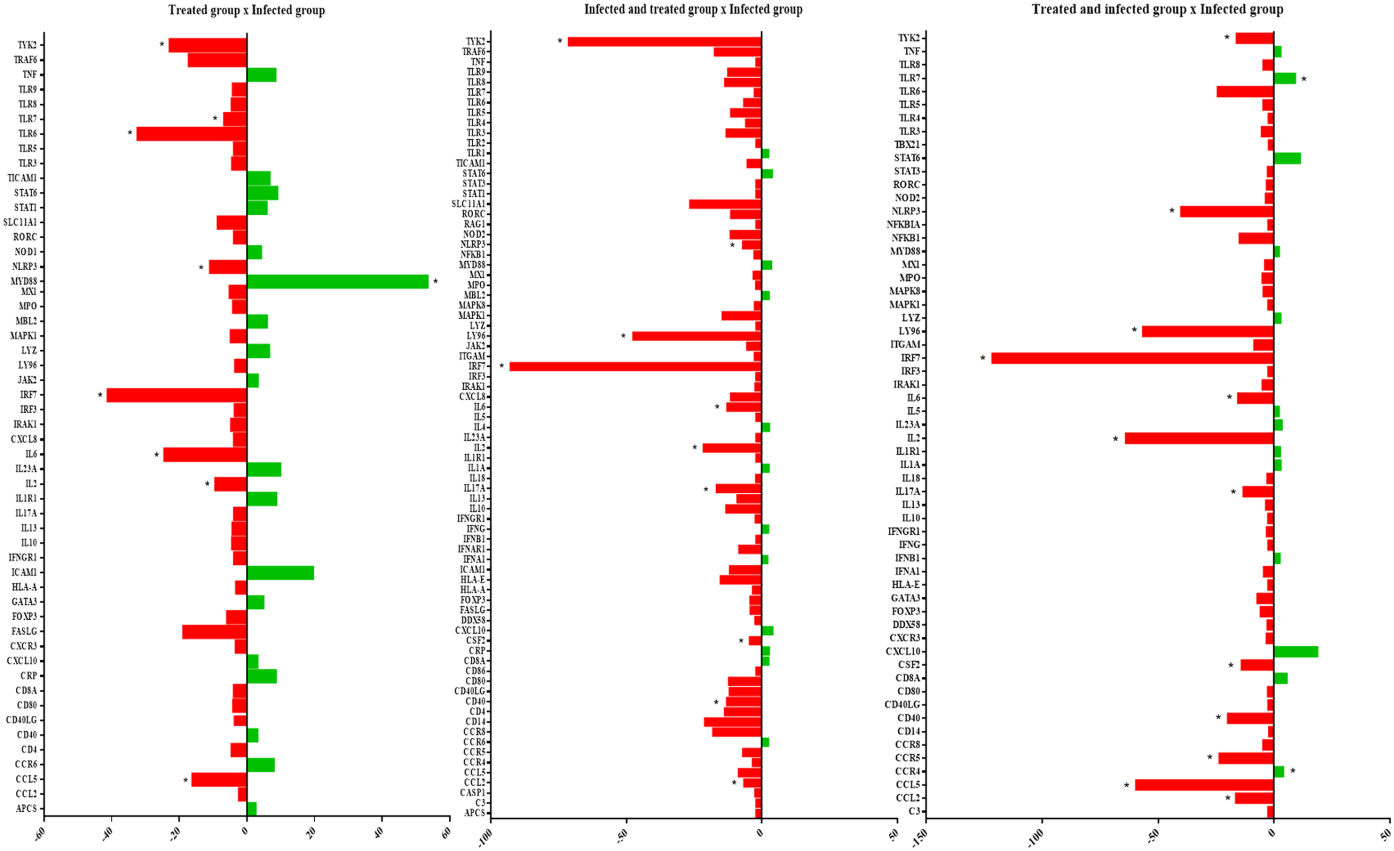
Comparison of the gene expression of the treated group with the association of β-caryophyllene and docosahexaenoic acid (50 + 50 mg/dL) with the infected-only group with methicillin-resistant *Staphyloccocus aureus* (**A**), the group infected with methicillin-resistant *Staphyloccocus aureus* and the group treated with BCP-DHA (**B**) and the group treated with BCP-DHA and infected with methicillin-resistant *Staphyloccocus aureus* (**C**). Data are expressed as mean ± DPM. *p < 0.05.

Figure 2 illustrates the comparative analysis of the treated group with the infected group with regard to innate and adaptive immunity genes. The innate immunity genes CCL5, IL-2, and IL-6, which are related to the synthesis of pro-inflammatory cytokines IRF7, NLRP3, TLR6, TLR7, and TYK2, associated with standard recognition and the defense response of the organism, were under-expressed with BD treatment. MYD88, the defense response gene, was hyper-expressed. Comparison between the infected and treated groups with the group that was only treated revealed that there was no hyperexpression of any gene with statistical significance; however, pro-inflammatory cytokine activation genes such as CCL2, CSF2, IL-2, IL-6, and IL-17A, and those involved in the standard recognition and defense response to viruses and bacteria such as CD40, IRF7, LY96, NLRP3, and TYK2 were under-expressed. The comparison between the treated and infected versus infected-only groups showed significant under-expression of CCL2, CCL5, CSF2, IL-2, IL-6, IL-17A, CD40, IRF7, NLRP3, LY96, and TYK2, as in the previous groups, as well as in the CCR5-Th1 adaptive response gene marker, and hyperexpression of TLR7 and CCR4-Th2 markers.

**Figure 3:**
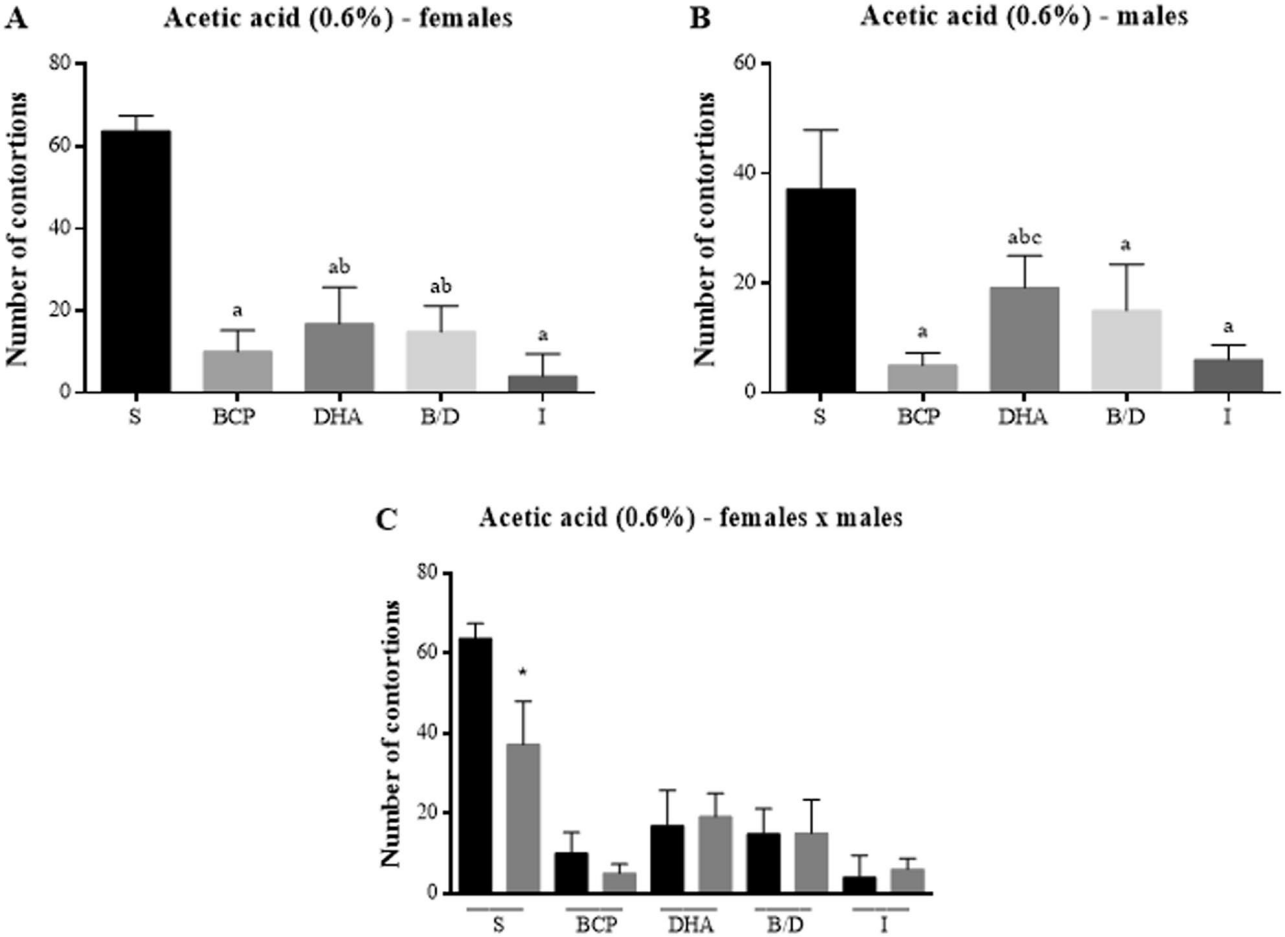
Number of abdominal writhing induced by acetic acid in Balb-C mice. (**A**) Female mice. (**B**) Male mice. (**C**) Comparison between sexes. S = saline; BCP = beta-caryophyllene; DHA = docosahexaenoic acid; B/D = BCP and DHA combination; I = indomethacin. Data are expressed as mean ± DPM. a *p* < 0.05 vs. S; b *p* < 0.05 vs. I; c *p* < 0.05 vs. BCP; *p < 0.05.

In the acetic acid test, there was a lower number of abdominal writhing among females in the indomethacin-treated group, followed by the isolated BCP and BD association groups (Fig. 3A). In males, there was statistical significance in all test groups regarding the control, and the BCP group presented similar results to indomethacin, but with no statistical difference between the groups (Fig. 3B). When comparing the behaviors between the sexes of the treated groups, there was an observable statistical difference only in the comparison of the saline control group (Fig. 3C).

**Figure 4:**
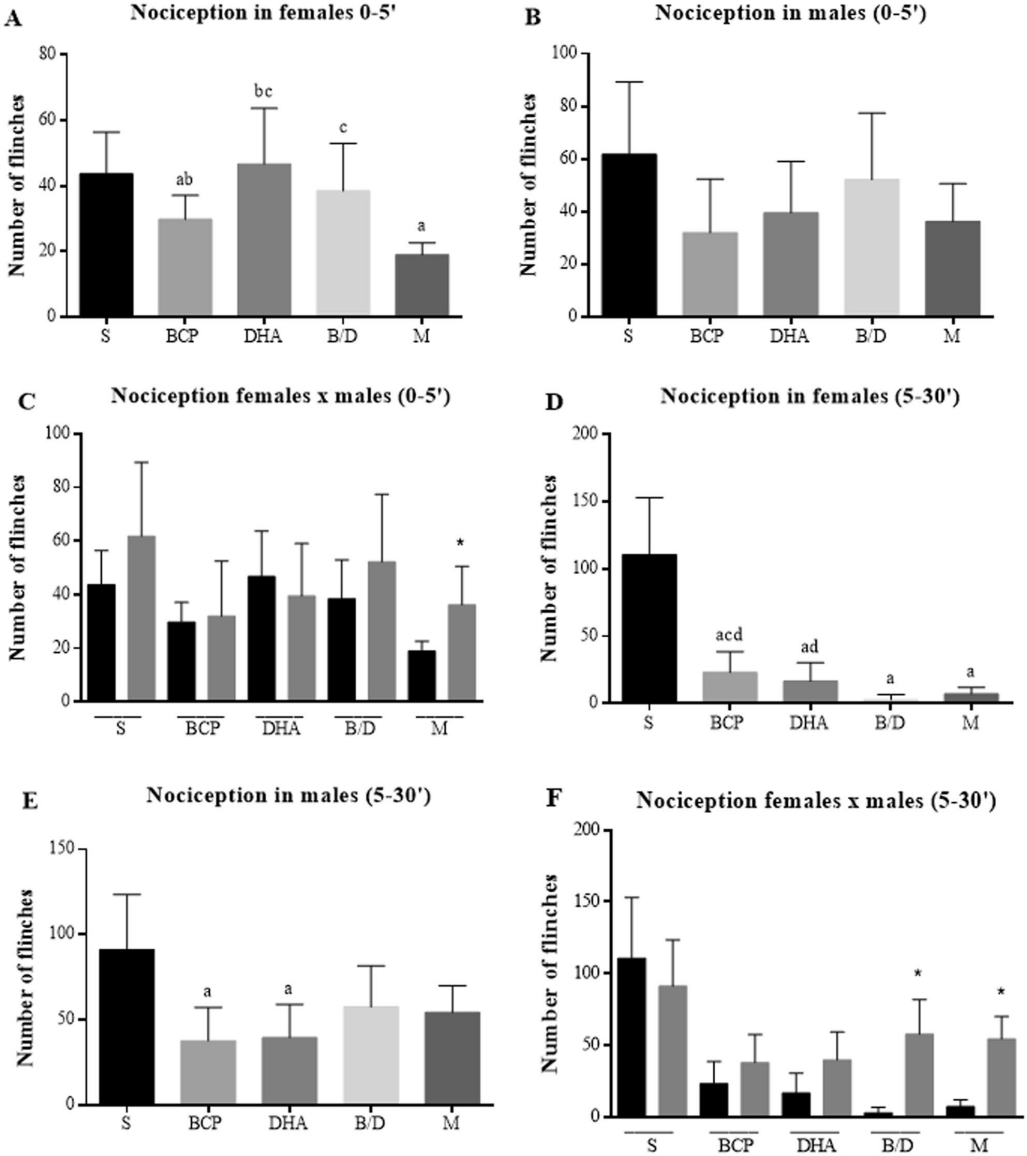
Number of flinches exhibited by Balb-C mice after intraplantar formalin administration (1.5%). (**A**) Females in 0–5 min period. (**B**) Females in 530 min period. (**C**) Males in 0–5 min period. (**D**) Males in 5–30 min period. (**E**) Comparison between sexes in the period 0–5 min. (**F**) Comparison between sexes in the period 0–5 min. S = saline; BCP = beta-caryophyllene; DHA = docosaehexanoic acid; BD = BCP and DHA combination; M = morphine. Data are expressed as mean ± DPM. a *p* < 0.05 vs. S; b *p* < 0.05 vs. M; c *p* < 0.05 vs. BCP; d *p* < 0.05 vs. BD; *p < 0.05.

In the neurogenic phase of the formalin test (first 5 min), the group treated with BCP demonstrated a significant reduction in the number of flinches in females compared to the saline group, which was not observed in the other treatment groups (Fig. 4A). There was no statistical difference in the male evaluations during the same period (Fig. 4B). In the comparison between sexes (Fig. 4C), it was observed that the nociception levels of the males were higher than those of females, but a statistical difference was found only in the group treated with the standard analgesic. When observing the inflammatory phase (5–30 min), the three treatments caused a significant reduction in the number of paw flinches in females, and the group treated with the BCP and DHA combination presented a similar result to that of the group that received morphine (Fig. 4D). In males, the best results achieved in the 5–30 min period were from the groups treated separately with BCP and DHA (Fig. 4E), but they showed a higher number of flinches when compared to the females, with proven statistical significance in the group treated with the BCP and DHA combination and with morphine (Fig. 4F).

**Figure 5:**
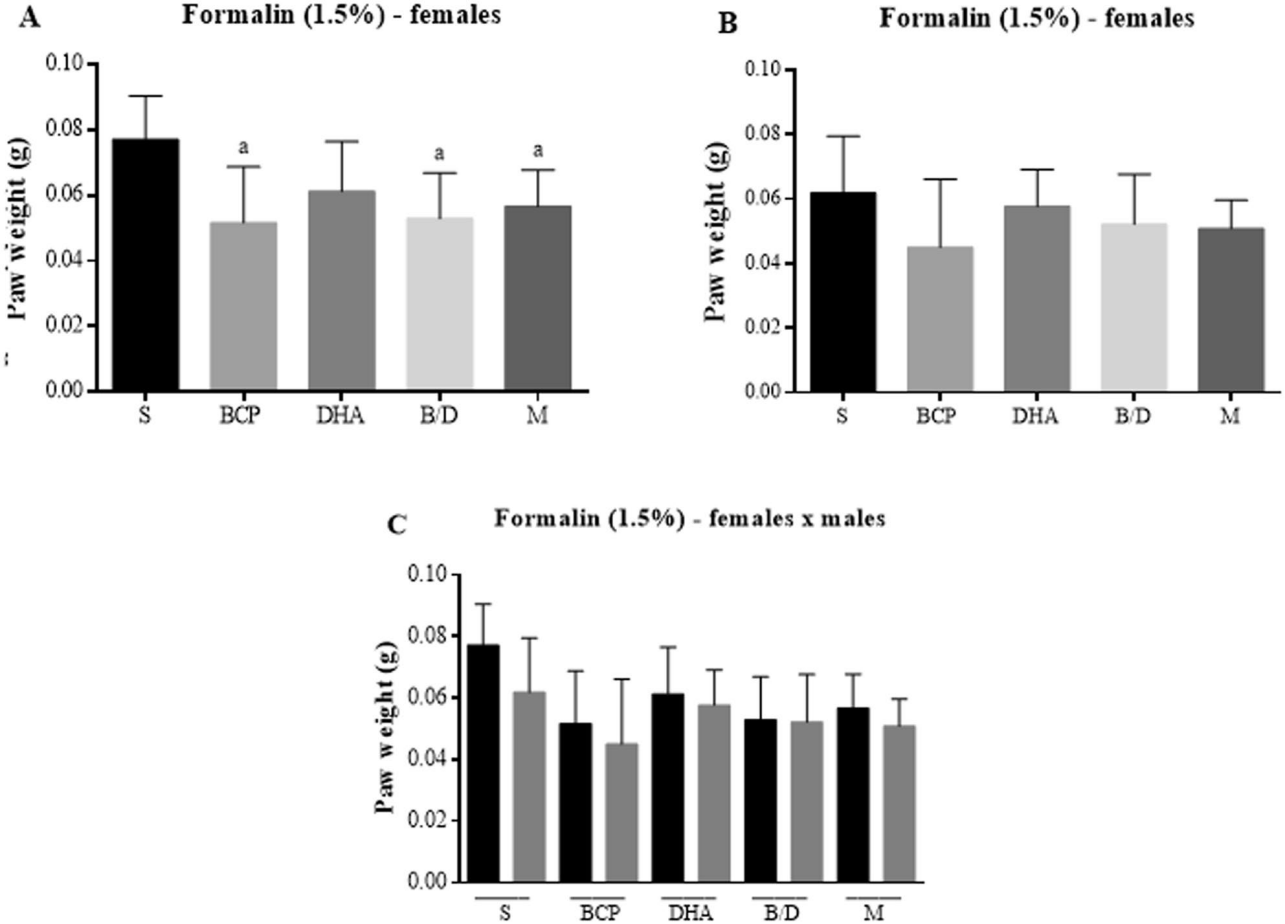
Paw weights of Balb-C mice that received formalin intraplantar injection (1.5%). (**A**) Female mice. (**B**) Male mice. (**C**) Comparison between sexes. S = saline; BCP = beta-caryophyllene; DHA = docosaehexanoic acid; B/D = BCP and DHA association; M = morphine. Data are expressed as mean ± DPM. a *p* < 0.05 vs. S.

Figure 5 shows the paw weight results. Only female rats had reduced paw edema in the isolated BCP groups and their association with DHA. The comparison between sexes also showed no statistically significant differences.

**Figure 6:**
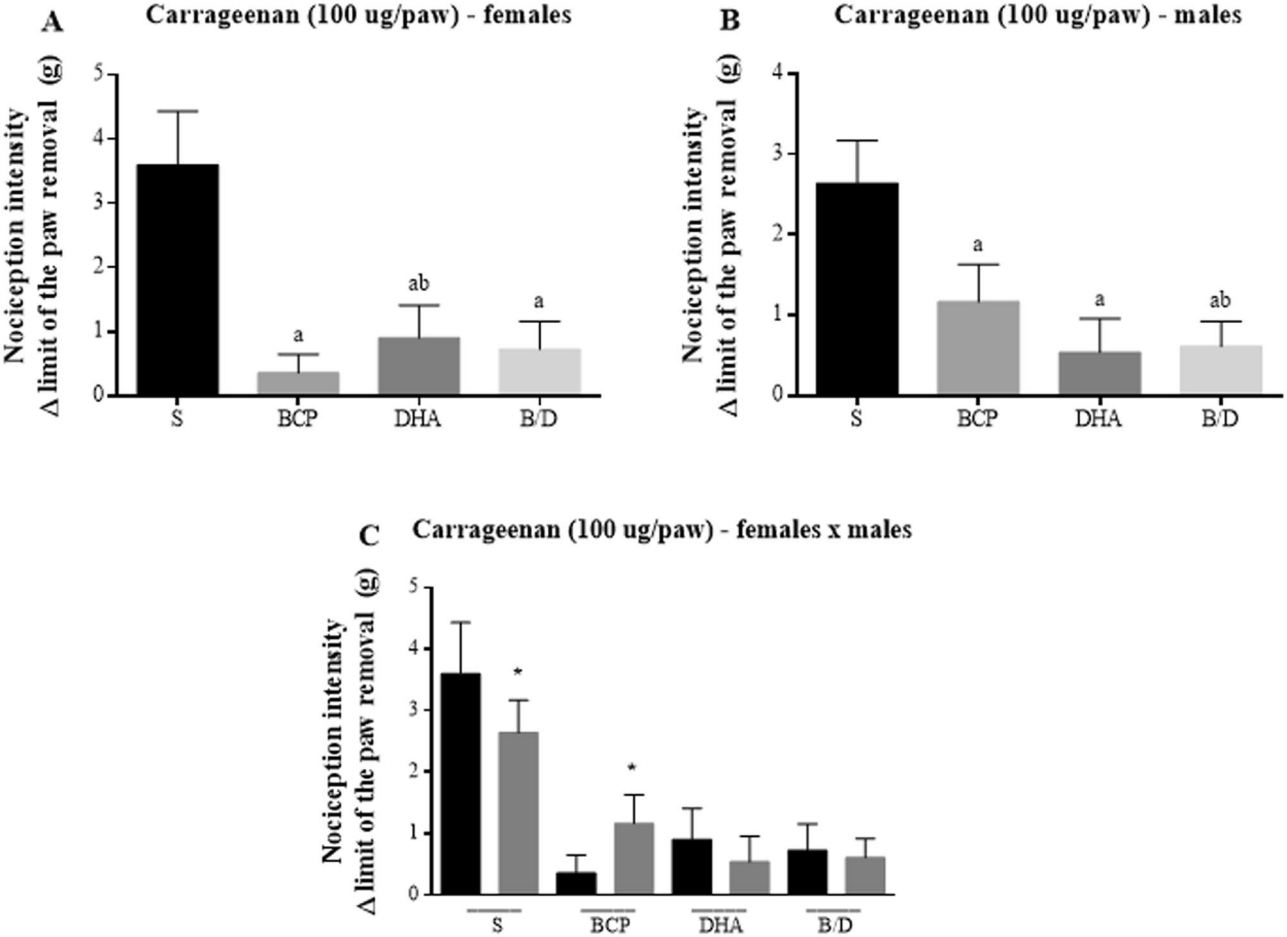
Nociception intensity by ∆ limit of the paw removal after Carrageenan injection (100 µg) in Balb-C mice. (**A**) Female mice. (**B**) Male mice. (**C**) Comparison between sexes. S = saline; BCP = beta-caryophyllene; DHA = docosahexaenoic acid; BD = BCP and DHA association. Data are expressed as mean ± DPM. a *p* < 0.05 vs. S; b *p* < 0.05 vs. BCP; * *p* < 0.05.

The three treatments significantly reduced the intensity of mechanical nociception measured using the von Frey method in female and male mice, with the best results obtained in the BCP groups in females and DHA groups in males (Fig. 6A,B). As shown in Fig. 6C, males were more sensitive to hypernociception in the BCP-treated group, with statistical significance, whereas females were more sensitive in the DHA-treated and B/D-associated groups, but without statistical significance.

## Discussion

Natural products have long been used in medicinal preparations and studied in the development of medicines, such as analgesic, antimicrobial, antitumor, and anti-inflammatory treatment^16^. The mechanisms of inflammation and infection constitute important research focuses, as they involve several cellular processes, such as chemotaxis, phagocytosis, immune response, differentiation, and apoptosis^17^.

In the MRSA infection model in our study, the cytokine dosage showed that treatment with BCP-DHA significantly reduced the levels of GM-CSF, TNFα, IL-1, IL-6, and IL-12. The synthesis of IL-10 was higher in the previously treated and later-infected groups and lower in the infected and later-treated groups. GM-CSF is a hematopoietic growth factor known for its ability to form granulocyte and macrophage colonies, with its receptor activation, the GM-CSFr, as an initiator to an intracellular signaling cascade that includes signal transducer activation and transcription activator 5 (STAT5), Janus kinase (JAK), MEK/ERK pathway, phosphatidylinositol 2-kinase (PI3K), and NF-kB^18^ acting on myeloid cells in inflammation and autoimmune diseases^19^. In the aging model and memory analysis used by Lindsey et al.^20^, BCP had no effect on this cytokine. In addition, the presence of DHA in the medium containing GM-CSF resulted in depletion of the hematopoietic stimulant factor in a cystic fibrosis model, showing that the deficiency of AGPI and its derived inflammation resolution is limiting to the unregulated inflammation present in the disease^21^.

TNF-α has broad functional relevance, mainly in terms of stimulation of cellular apoptosis through Faz and caspases, and pro-inflammatory responses through NF-ĸB activation^22^. In a previous study, an increase in TNF-α in the group treated with the BCP-DHA combination was found in the sepsis model, which may be related to its susceptibility to stimulation by COX inhibitors. Both natural products develop this role^23^. We now find divergent results with decreased concentrations of this cytokine using an in vitro model. The main IL-1 β secretors are hematopoietic cells such as monocytes, macrophages, microglia, Kupffer cells, and dendritic cells after activation by damage-associated molecular patterns (DAMPs)^22^. It is a cytokine that is closely linked to inflammation and fever, and its synthesis is associated with inflammasome activation^23^. The pro-inflammatory characteristic also represents IL-6, a cytokine involved in immune responses, hematopoiesis, bone metabolism, the development of autoimmune diseases, bacterial infections, and metabolic side effects^22,24^. In vitro research suggests that the BCP agonist activity on CB2r is the main mechanism responsible for the inhibition of pro-inflammatory pathways and cytokine synthesis, such as IL-1β, IL-6, and TNF-α^25,26^. Inhibition of IL-1β and TNF-α by DHA has also been described in the literature, with emphasis on greater PPAR-γ activation and NF-ĸB inhibition^27,28^. The IL-12 cytokine family is involved in modulating the behavior of T-cell populations and targeting immune responses in various diseases owing to their pro-inflammatory role^29^. They are link between innate and adaptive immunity, mediating the differentiation of naive CD4 T cells into T helper subtypes, and regulating the functions of different cell effectors^30^. A reduction in IL-23, a cytokine of the IL-12 family, was observed in the animal senescence model treated with BCP^19^, and DHA inhibited dendritic cell maturation, reducing IL-12 family levels (IL-12p70, IL-23, and IL-27)^31^.

Following a regulatory pathway, the IL-10 family plays an important role in regulating the immune response during host defense, autoimmune and inflammatory diseases, and cancer. IL-10 acts primarily with immunosuppressive functions on leukocytes, whereas other members of the family act preferentially on epithelial cells, where they control defense mechanisms against viral, bacterial, and fungal infections, protect tissue integrity, and promote repair and regeneration^32,33^. DHA supplementation promotes the reduction of IL-10 synthesis by conferring cardioprotection in a myocardial injury model^34^. A study with the essential oil of *Polygonum minus* identified BCP as one of its components and used it as a positive control in the cisplatin-induced hepatotoxicity model, demonstrating a reduction in the concentration of IL-10 and, therefore, hepatoprotection^35^. Therefore, the reduction in our study demonstrates protection against exacerbation of inflammation during the infectious process.

**Figure 7:**
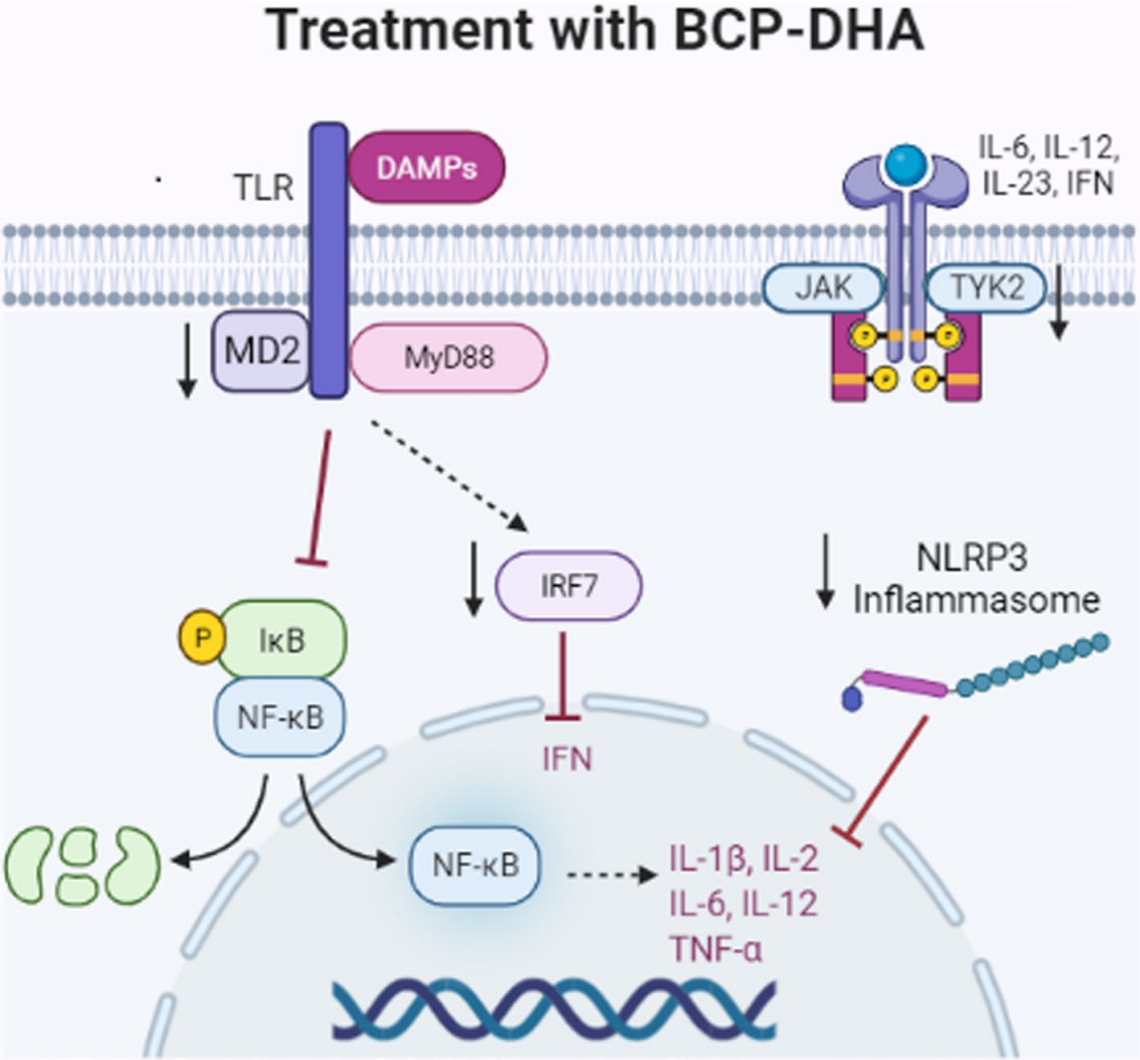
Anti-inflammatory characterization of the BCP-DHA treatment in the face of MRSA infection. The recognition of *S. aureus* by toll-like receptors present in the cell membrane initiates an intracellular signaling cascade that results in the dissociation of the NF-κB/Iκb complex, NF-κB translocation to the cell nucleus, and stimulation of the synthesis of pro-inflammatory cytokines. BCP-DHA treatment acts through inhibiting different pathways that result in the activation of NF-κB, negatively regulating the expression of the MD2, IRF7, NLRP3, and TYK2 genes, which culminates in lower synthesis and acting of pro-inflammatory cytokines.

Many anti-inflammatory effects occur in the body by altering the expression patterns of innate immune genes. In our study, we observed that treatment with BCP-DHA, regardless of the presence of infection, negatively regulated the gene expression of IL-2, IL-6, IRF7, NLRP3, and TYK2, acting directly in NF-ĸB inhibition and the synthesis and activity of pro-inflammatory cytokines (Fig. 7). Within the interferon (IFN) signaling pathway, IRF7 has been recognized as the major signaling regulator of IFN type 1 and is involved in the control of excessive inflammation and autoimmunity^36,37^. In a silica-induced lupus model, DHA inhibited the expression of IRF7 and other IFN-associated genes involved in the inflammatory response^38^. In the inflammatory pathway, NLRP3, a component of the inflammasome cytosolic multiprotein complex generated in bone marrow-derived macrophages, stimulated by microbial agents, is also involved. The NLRP3 inflammasome plays a critical role in innate immunity by stimulating active caspase-1 synthesis, which converts IL-1β and IL-18 precursors into their biologically active forms in a sequence of inflammatory responses that leads to pyroptosis^39^. Inhibitory activity of BCP on NLRP3 was observed in a gouty arthritis model in animals^40^ and of DHA on the NLRP3 inflammasome induced using silica inhalation. The role of BCP-DHA in the negative regulation of the non-receptor tyrosine kinase TYK2, whose activation regulates the signal transduction pathways of IL-12, IL-23, and IFN type 1 receptors, has brought novelty to the literature, each of which is implicated in the pathogenesis of various inflammatory diseases^42,43^.

Under infectious stimuli, we observed that there was also an under-expression of genes related to inflammatory pathways, such as CCL2, CSF2, CD40, and LY96. CCL2 is a monocyte chemoattractive protein expressed in endothelial cells, smooth muscles, fibroblasts, astrocytes, T cells, and tumor cells after an inflammatory stimulus (e.g., IL-1β, IL-6, TNFα, LPS, and GM-CSF)^44^. Treatment with DHA led to rapid and limited CCL2 expression in a myocardial injury model, indicating restricted inflammation^34^. In a dextran sulfate-induced colitis, there was negative regulation of CCL2 expression after treatment with BCP^45^. CSF2, which encodes the GM-CSF cytokine, is directly involved in the conversion of the M1 to M2 phenotype in macrophages and plays a crucial role in inflammation^46^. CD40 belongs to the TNFα family and therefore has pro-inflammatory and pro-thrombotic effect^47^. As mentioned previously, BCP and DHA act by negatively regulating the expression of GM-CSF and TNF-α cytokines. LY96, or myeloid differentiation factor 2 (MD2), is a co-receptor that, by identifying the LPS, is coupled to TLR4, forming the LPS-MD2-TLR4 complex, and promotes the recruitment of myeloid differentiation protein (Myd88), activating the MAPKs and NF-kB, resulting in stimulation of the synthesis of pro-inflammatory cytokines^48^. The literature describes the agonist effects of BCP on CB2r, including inhibition of the CD14/TLR4/MD2 complex as a potent pathway for regulating inflammation^49,50^. It has recently been suggested that DHA inhibits the formation of this complex, preventing the intracellular inflammatory signaling cascade by competing with LPS for the binding site^51^.

In the treated-only group, positive regulation of Myd88 was observed; this is a downstream TLRs adaptor protein, with the exception of TLR3, which is critical for signal transduction in the pathway that results in NF-ĸB^52^ activation. Although studies have shown that BCP and DHA promote the suppression of Myd88 expression^40,53,54^, the present study showed the opposite effect. In the treated and later infected groups, there was increased expression of TLR7 and CCR4, a chemokine receptor predominantly expressed by Th2 cells and related to inflammatory diseases and T cell neoplasms, such as leukemias and lymphomas^55–57^.

The above observations show the relevant antinociceptive potential of the BCP-DHA combination with results compatible with the first-choice drugs in the nociception tests performed, as well as its anti-inflammatory ability by significantly inhibiting the cytokine levels of pro-inflammatory GM-CSF, TNFα, IL-1, IL-6, and IL-12. The present study showed the negative regulation of crucial genes in the inflammatory process, such as IL-2, IL-6, IRF7, NLRP3, CCCL2, CSF2, CD40, and LY96, whose pathways result in the inhibition of NF-ĸB dissociation and in the synthesis of the pro-inflammatory cytokine/acting. TYK2 inhibition through treatment using BCP-DHA was also shown for the first time.

In the present study, we showed that BCP-DHA association and isolated BCP presented the best antinociceptive responses in the acetic acid-induced writhing test in both sexes. The formalin test demonstrated a significant reduction in the number of flinches in females treated with BCP during the neurogenic phase. In the inflammatory phase, a better treatment result was observed with BCP-DHA in relation to morphine in females, and better results were seen for isolated compounds in males. Reduced paw edema was also observed in females of the mentioned groups. Both isolated and combined BCP and DHA promoted a significant reduction in mechanical nociception in intraplantar Cg injections in the von Frey test in females and males. Our findings of better pain responses of isolated BCP and BCP-DHA are similar to those found by Fiorenzani et al.^14^ with the formalin test in the first study that associated these substances, except that they worked only with male rats, and the present study used both sexes.

Recent studies have shown that differences between sexes contribute to individual differences in pain perception and treatment, but the specific mechanisms of disparity remain inconclusive. It is possible that interactions between biological, sociocultural, and psychological factors, mainly sex hormones (androgens and estrogens), the endogenous opioid system, and genetic factors, are involved in the process^17,58^^.^ Experimental pain models highlight the pronociceptive role of estrogen in male rats that received intracerebroventricular estradiol injections, as well as the antinociceptive effect on neuropathic pain in mice^59^. Male and female rats reacted differently to structural and functional changes related to pain induced by sciatic nerve ligation, with males showing gradual allodynia reduction and complete recovery, whereas allodynia and gliosis in females lasted for 4 months, suggesting that male results are associated with testosterone and that females are sensitive to changes in serum testosterone^60^. Overall, women appear to have greater pain sensitivity and better pain responses than men. In addition, studies suggest that there are differences in responses to pharmacological and non-pharmacological treatments, depending on the type of treatment and the characteristics of the pain^58^.

Nociception, as a part of the inflammatory process, can be neurogenic or inflammatory, and its stages depend on the duration of the process, as well as immunological factors^61^. The antinociceptive effect of BCP was described in the acetic acid test, with a result similar to that of indomethacin^62,63^ in neuropathic pain in a model of diabetes induced by streptozotocin in mice^64^ and in study that used a partial ligation model of the sciatic nerve in mice^65^. This may be due to its binding to the CP55.940 site of cannabinoid receptor type 2 (CB2r), which leads to inhibition of microglial activation during neuropathic pain, causing analgesia through supraspinatus, spinal, and peripheral CB2r activation^66^. The antinociceptive activity of DHA in thermal and chemical pain models is abolished in the presence of naloxone, indicating that it acts in the opioid pathway as a receptor antagonis^67^. Therefore, the potential antinociceptive effects of this combination were highlighted in this study. In addition, our results strongly suggest a possible new therapeutic alternative in the context of pain and inflammation, requiring further study to improve the doses and routes of administration.

## Materials and methods

### Ethical and legal aspects

This study was conducted at the Microbiology and Immunology Laboratory of the Multidisciplinary Health Institute of the Bahia Federal University (IMS/UFBA) after approval was obtained from the Ethics Committee on Research on Human Beings (CEPSH) and the Ethics Committee on Animal Use (CEUA) of the Bahia Federal University, under protocols nos. 2,791,699 and 060/2018, respectively. All procedures were initiated only upon approval by the latter, and after the donors provided informed consent. The 80 strains of methicillin-resistant *S. aureus* (MRSA) used in the study were isolated from the nasal swabs of healthy children, aged between 6 and 8 years, from daycare centers located in Vitória da Conquista, Bahia, Brazil. The strain was obtained from another study approved by the IMS/UFBA CEPSH (protocol no. 08731912.5.0000.5556).

### BCP and DHA

BCP and DHA marketed by Sigma-Aldrich® were used. DHA was diluted in 10% ethanol, as recommended by the manufacturer. Doses used were defined based on a previous study^15^.

### Human peripheral blood monocyte isolation and infection

Peripheral blood collections were performed on three male participants, older than 18 years, who agreed to participate in the study and signed the free informed and clear consent form (TCLE). Previously, the volunteers had been subjected to an evaluation of their general health status through laboratory tests because alterations in these parameters can interfere with the cell culture and immunological response. On the day of the experiment, blood samples (20 mL) were kept at room temperature and processed within 2 h of collection. PBMCs were separated using centrifugation in with a Ficol column (200×*g* for 10 min at 4 °C) and resuspended in RPMI 1640 medium with 10% fetal bovine serum (FBS, GIBCO BRL). After the evaluation of the quantity and adequate viability of the cells, monocytes were inoculated and/or treated with BPC and DHA^8,68,69^.

Monocytes were previously grown in polystyrene bottles using specific media for each cell type (incubation: 37 °C with 5% CO^2^). Upon reaching an ideal confluence of approximately 70% (~ 10^6^/mL), the cells were infected with *S. aureus* (1 × 10^6^) and incubated with BCP-DHA (Sigma Aldrich) for 30 min at 37 °C (10^–10^ × 10^–7^ M)^72^. Negative controls, containing infected cells without treatment or untreated cells, were also analyzed. All experiments were performed in triplicate. After this process and the determined time of evaluation had passed, the supernatant of each culture was collected and frozen at − 70 °C for later cytokine production. For gene expression testing, cell suspensions were transported to microtubes for RNA extraction^70,71^.

Cytokine dosage in the cell culture supernatant was measured using flow cytometry with a Human Th1/Th2/Th17/Th22 13plex – Flow Cytomix commercial kit (eBioscience: Bender Med Systems Gmbh) to quantify GM-CSF, IL-1β, IL-6, IL-10, IL-12, and TNFα, according to the procedures described by the manufacturer’s instructions. FlowCytomix Pro software was used for data analysis, according to the manufacturer’s guidelines.

The gene expression of inflammatory markers was evaluated using the PCR array methodology. The mRNA of the macrophage samples was extracted using TRIzol® LS (Life Technologies™) and following the manufacturer’s protocol. cDNA was obtained using retro-transcription (RT) of mRNA with the SuperScript® IV Reverse Transcriptase kit with the addition of complementary oligonucleotides to the poly-A mRNA tail (Oligodt) and an RNase inhibitor. The cDNA obtained was subjected to analysis using the *Human Innate & Adaptive Immune Responses* PCR Array (Qiagen-AS Bioscience) for the evaluation of 84 genes involved in host response associated with inflammation and bacterial infection. These genes are involved in innate immunity, adaptive, humoral, and inflammatory and defense responses to bacteria and viruses.

### Animals

A total of 168 mice of the Balb-C lineage, aged between 6 and 8 weeks, from the Multidisciplinary Center for Biological Research in the Animal Science Laboratory of Campinas State University (CEMIB/UNICAMP) were used. As recommended by the American Society for Microbiology, we analyzed males and females (84 animal from each sex). The animals were kept in the IMS/UFBA Vivarium for mice (six animals per cage) under controlled conditions of light (lights on from 7a.m to 7p.m) and temperature (23 ± 3 °C) and had free access to water and food.

### Test of abdominal writhing induced by acetic acid

Five experimental groups of six mice (BALB/c) each, fasted for 12 h, were used per sex. Before the test (24 h), the following was administered subcutaneously: 0.9% NaCl solution (10 mg/kg), indomethacin (10 mg/kg), BCP (5 mg/kg), BCP + DHA (2.5 mg/kg each), and DHA (5 mg/kg). The next day, the animals received an injection of acetic acid (0.6%; 10 mL/kg, intraperitoneal). The produced abdominal wall writhing, followed by trunk turning and hind limb extension, was counted for 20 min as an indication of nociception^72^.

### Test of nociception induced by formalin intraplantar injection

Five experimental groups of six mice (BALB/c) each, fasted for 12 h, were used per sex. Animals were subcutaneously administered the following 24 h before the test: 0.9% NaCl solution (10 mg/kg), morphine (5 mg/kg), BCP (5 mg/kg), BCP + DHA (2.5 mg/kg each), and DHA (5 mg/kg). The next day, a formalin solution was administered at a 1.5% concentration (formaldehyde, intraplantar, 20 μL) to the right posterior paws of the animals. The number of flinches was quantified over 30 min as an indicator of nociception. The first 5 min determined the response to pain of neurogenic origin, whereas the final 15 min determined the response to pain of inflammatory origin. In this test, edema in the right paw was also measured, and the difference between the weight of the right paw and the weight of the left paw was calculated by weighing the paws^72^.

### Hypernociception evaluation to test increasing pressure in the paw

Von Frey filaments were used to determine mechanical nociception^73^. The animals were placed in acrylic boxes with mesh network floors consisting of non-malleable wire for 30 min before the experiment to adapt to the environment. The test consisted of producing pressure on the paws of the animals with a hand force transducer adapted with a tip (0.8 mm^2^ tip diameter, Von Frey electronic, Insight Equipment©, Brazil). The stimulus was automatically stopped when the animal presented a response characterized by flinching in response to the stimulated paw and the intensity was recorded. The interval between two consecutive tests on the same paw was at least 1 min, totaling six trials per animal. The maximum force applied was 50 g. Four experimental groups of six mice (BALB/c) each, fasted for 12 h, were used per sex. Animals were subcutaneously administered the following 24 h before the test: 0.9% NaCl solution (10 mg/kg), BCP (5 mg/kg), BCP + DHA (2.5 mg/kg each), and DHA (5 mg/kg). The next day, carrageenan (100 μg/paw, i.pl.) was injected into the ventral surface of the right hind paws of the animals, and after 3 h, the von Frey test was performed. The hypernociception intensity was used to quantify the change in pressure (Δ of reaction in grams) obtained by subtracting the value observed before the experimental procedure (0 h) from the reaction value.

### Statistical analysis

Statistical analysis was performed using the GraphPad Prism software (version 6.0; GraphPad Software, San Diego, CA, USA). The comparisons performed in the different experiments were determined using individual variation or variation of error (s2), the analysis of the Shapiro–Wilk normality test, and the Mann–Whitney test by pairs, because the data did not present a normal distribution. The results are expressed as mean ± standard deviation of the mean (DPM). Statistical differences were considered significant at *p* < 0.05, using a 95% confidence interval.

### Ethics approval and consent to participate

All methods in this current study are reported in accordance with ARRIVE guidelines. All experiments with mice also were conducted in accordance with internationally accepted principles for the use and care of laboratory animals, as established in the Brazilian guideline for the care and use of animals in teaching or scientific research activities (DBCA) related to principles of conduct that ensure the care and ethical management of animals used for scientific or teaching purposes, and carried out after approval by the Ethics Committee on the Use of Animals (CEUA) of the Instituto Multidisciplinar em saúde of Universidade Federal da Bahia (project number 60/2018).

The study also was developed after approval by the Human Research Ethics Committee of the Federal University of Bahia, Multidisciplinary Institute for Health (CAAE: 79446117.5.0000.5556) and was carried out in accordance with The Code of Ethics of the World Medical Association (Declaration of Helsinki) for experiments involving humans. The strains used were obtained from other studies after approval by the Ethics Committee of Research with Human Beings of the Multidisciplinary Health Institute campus Anísio Teixeira (CAAE nº 08730012.4.0000.5556) and 08731912.5.0000.5556 (nasal strains). For nasal samples, informed consent was obtained from the parents or guardians.

## Data Availability

The datasets used and/or analyzed during the current study are available from the corresponding author upon reasonable request.
